# Feasibility of different meningioma delineation approaches on [^18^F]SiTATE PET/CT imaging

**DOI:** 10.1007/s12672-025-04079-6

**Published:** 2025-11-14

**Authors:** Sophie Carina Kunte, Lena M. Unterrainer, Simon Lindner, Adrien Holzgreve, Wolfgang G. Kunz, Michael Winkelmann, Alexander Nitschmann, Klaus Jurkschat, Carmen Wängler, Björn Wängler, Ralf Schirrmacher, Claus Belka, Niklas Thon, Christian Schichor, Peter Bartenstein, Marcus Unterrainer

**Affiliations:** 1 Department of Nuclear Medicine, LMU University Hospital, LMU Munich, Marchioninistr. 15, 81377 Munich, Germany; 2 Bayerisches Zentrum für Krebsforschung (BZKF), partner site Munich, Munich, Germany; 3 Ahmanson Translational Theranostics Division, David Geffen School of Medicine at UCLA, Los Angeles, CA USA; 4 Department of Radiology, LMU University Hospital, LMU Munich, Munich, Germany; 5 Department of Radiation Oncology, LMU University Hospital, LMU Munich, Munich, Germany; 6 Fakultät für Chemie und Chemische Biologie, Technische Universität Dortmund, Dortmund, Germany; 7 Biomedical Chemistry, Department of Clinical Radiology and Nuclear Medicine, Medical Faculty Mannheim of Heidelberg University, Mannheim, Germany; 8 Molecular Imaging and Radiochemistry, Department of Clinical Radiology and Nuclear Medicine, Medical Faculty Mannheim of Heidelberg University, Mannheim, Germany; 9 Department of Oncology, Division of Oncological Imaging, University of Alberta, Edmonton, AB Canada; 10 German Cancer Consortium (DKTK), Partner Site Munich, Munich, Germany; 11 Department of Neurosurgery, LMU University Hospital, LMU Munich, IE RADIOLOGIE, Munich, Germany; 12 DIE RADIOLOGIE, Munich, Germany

**Keywords:** Meningioma, [^18^F]SiTATE, Delineation, SSTR, SUV, Isocontour

## Abstract

**Background:**

Somatostatin receptor (SSTR)-targeted PET is valuable for meningioma imaging due to high SSTR expression. [^18^F]SiTATE, a novel tracer, is not only promising for imaging neuroendocrine tumors but also for meningiomas. Standardized delineation methods on [^18^F]SiTATE PET are lacking. This study correlates CT-based volumes with PET-based delineation approaches to identify a threshold for standardized [^18^F]SiTATE PET volume assessment.

**Methods:**

Patients with well-delineated, extraosseous meningioma on CT (≥ 1mL) who underwent [^18^F]SiTATE PET/CT were included. Volumes were assessed on contrast-enhanced CT and correlated with PET-based delineation approaches: (I) fixed SUV threshold, (II) isocontour thresholding relative to SUV_max_ (SUV%), and thresholds relative to (III) bone marrow (SUV_BM_), (IV) parotid gland (SUV_parotis_) and (V) pituitary gland (SUV_sella_).

**Results:**

19 meningiomas in 17 PET/CT scans (16 patients) were included. A fixed SUV of 4.0 (*r* = 0.783, *p* < 0.001) showed good correlation with CT volumes without skewed distribution on Bland-Altman-Plot analysis. Using isocontour-based thresholds, 45% SUV_max_ (*r* = 0.496, *p* = 0.031) showed the highest concordance. Best reference-based approaches were achieved by 150% SUV_BM_ (*r* = 0.859, *p* < 0.001), 250% SUV_parotis_ (*r* = 0.460, *p* = 0.047) and 70% SUV_sella_ (*r* = 0.819, *p* < 0.001). However, background-based approaches showed a trend towards overestimation of PET-volumes in larger meningiomas as assessed on Bland-Altman-Plot analyses. Uptake intensities of reference tissues (SUV_BM,_ SUV_parotis_ and SUV_sella_) were not inter-correlated (*p* > 0.05 each).

**Conclusion:**

A fixed SUV threshold of 4.0 showed strong agreement with CT-based volumes in well-delineated, extraosseous meningiomas which offers a simple, clinically applicable method without technical requirements. Reference tissue-based methods showed similar correlations but tended to overestimate volumes in larger lesions.

**Supplementary Information:**

The online version contains supplementary material available at 10.1007/s12672-025-04079-6.

## Introduction

 The novel ^18^F-labeled somatostatin-receptor (SSTR) targeting peptide, [^18^F]SiTATE (formerly known as [^18^F]SiFA*lin*-TATE), has been used for neuroendocrine tumor (NET) and meningioma positron emission tomography (PET) imaging [1–6]. In comparison to ^68^Ga-labeled peptides, [^18^F]SiTATE provides significant logistical advantages, e.g. due to the longer half-life and higher resolution of the PET images, and can be used for surgery or radiotherapy planning [7–9]. To provide best decision making regarding therapeutical management, it is crucial to know the exact extent of meningiomas. Especially in patients with intraosseous extension of meningioma, it can be challenging to determine the volume of the meningioma on conventional imaging. This may affect the therapy outcome, e.g. if the area for radiotherapy is assumed to be too small or too large [10]. Hence, there is a need to establish a method to precisely determine the extent of meningiomas to ensure proper inclusion in radiotherapy and surgery planning [11].

With this preliminary study we aimed at identifying the best approach for standardized, semi-automatic tumor delineation on [^18^F]SiTATE PET/CT. Therefore, we identified cases with unequivocal meningioma extent on morphological contrast-enhanced CT (computed tomography) imaging and compared the CT volumes (“ground truth”) with PET volumes derived from different thresholds for semiautomatic tumor segmentation.

## Methods

### Inclusion criteria

This retrospective study was approved by the institutional ethics committee of the LMU Munich (IRB 22–0353). Patients who underwent SSTR PET/CT imaging of meningiomas in our department as part of routine clinical practice, e.g. for treatment planning, were included. Criteria for inclusion in our study were (i) [^18^F]SiTATE PET/CT; (ii) patients with well-delineated meningiomas on CT (CT-derived volume ≥ 1 mL); (iii) no suspicion for intraosseous expansion; (iv) no local pretreatment.

### Radiopharmaceutical and imaging protocol

A mean activity of 163 ± 40 MBq [^18^F]SiTATE was injected intravenously in line after premedication with furosemide (20 mg intravenously) with previously reported radiosynthesis and administration procedures [8, 12]. Radiopharmaceutical administration was based on an individual patient basis according to the German Pharmaceuticals Act § 13(2b). Cranial PET was performed using a Biograph mCT scanner or a Biograph 64 PET/CT scanner (Siemens Healthineers Erlangen. Germany). The PET/CT scan was performed 84 min after tracer injection (mean; SD 25.4) which included a diagnostic contrast-enhanced CT scan (slice thickness 0.3 cm) in portal-venous phase (Imeron 350; 1.5 mL/kg body weight; Bracco Imaging. Milano. Italy). Images were reconstructed iteratively using TrueX (three iterations. 21 subsets) with Gaussian post-reconstruction smoothing (2 mm full width at half-maximum). Reconstruction parameters were identical across all scanners, all of which are EARL-accredited to ensure standardized quantitative performance.

### CT image analysis

For CT analysis the volume of the respective meningioma was manually delineated on a slice-by-slice manner by creating a region of interest (ROI) and visually verified. The volume of interest (VOI) was calculated by merging the ROIs into a VOI. A dedicated workstation was used (Hermes Medical Solutions. Stockholm. Sweden).

### PET image analysis

For PET analyses, an ellipsoid volume of interest (VOI) was created surrounding the meningioma. Off-target [^18^F]SiTATE-avid areas such as the pituitary gland were excluded and visually verified to avoid erroneous results. Different approaches for volumetric delineation of the respective meningioma were applied on the respective VOI and correlated with the CT-derived reference volume; the following approaches were used:

I) fixed SUV thresholds: SUV 15.0; SUV 10.0; SUV 5.0; SUV 4.5; SUV 4; SUV 3.5; SUV 3.0 and SUV 2.5.
II) isocontour thresholding relative to SUV_max_ (SUV%): 10.0%; 15.0%; 20.0%; 25.0%; 30.0%; 35.0%; 40.0%; 45.0%; 50.0% and 55.0%.
III) thresholds relative to bone marrow (SUV_BM_), parotid gland (SUV_parotis_) and intrasellar pituitary gland (SUV_sella_).

For thresholds relative to SUV_BM_, a cubic 10 × 10 × 10 mm reference ROI was placed in the third cervical vertebra. The following threshold values were applied: 400.0%; 375.0%; 350.0%; 200.0%; 150.0% SUV_BM_ and SUV_BM_ minus 10%. 30%. 40.0%; 50.0%; 55.0%; 60.0%.

For thresholds relative to SUV_parotis_, a cubic 10 × 10 × 10 mm reference ROI was placed centrally in the parotis. The following threshold values were applied: 400.0%; 375.0%; 350.0%; 250.0%; 200.0%; 150.0% SUV_parotis_ and SUV_parotis_ minus 40.0%; 60.0%; 70.0%; 80.0%.

For thresholds relative to SUV_sella_, the mean SUV within a cubic 10 × 10 × 10 mm reference ROI placed in the pituitary gland was extracted, and the following values were used as thresholds for meningioma delineation: 125.0% SUV_sella_ and SUV_sella_ minus 30.0%; 35.0%; 40.0%; 50.0%; 60.0%; 70.0% and 80.0%.

Additionally, individual backwards thresholding was done: for every single meningioma, fixed threshold values were adjusted to achieve the identical PET- and CT-derived volume.

Analysis was performed as a consensus reading. A dedicated workstation (Affinity 1.1.4. Hermes Medical Solutions. Stockholm. Sweden) was used.

### Statistical analyses

Statistical analyses were performed using GraphpadPrism (version 9.4.0). Values are presented as mean ± standard deviation (SD) or median (range), depending on the results of the Shapiro–Wilk test for normality. Correlations between CT- and PET-derived volumes using different thresholds were assessed by either Spearman or Pearson correlation coefficients, chosen according to the normality of the data as determined by the Shapiro–Wilk test. The coefficient of variation (CoV) was used as standardized measure of dispersion of a probability distribution (ratio of the standard to the mean). Results were visualized using scatter plots and Bland-Altman plots. Statistical significance was defined as a two-sided p-value < 0.05.

## Results

### Patients

The mean age was 56.2 years (SD 14.9) (Supplementary Table). Lesions (*n* = 19) were located as following: frontal (4/19), cerebellar tentorium (3/19), parafalcine (3/19), falcine (2/19), frontobasal (1/19), frontoparietal (1/19), olfactory nerve (1/19), parietal (1/19), posterior sagittal sinus (1/19), temporal (1/19), temporoparietal (1/19).

### CT image analysis

The median CT-derived meningioma volume was 2.2 mL (range 1.1–6.1 mL).

### Volumetric correlation of different delineation approaches

The following Tables 1, 2 and 3 show the results of the correlation of PET volumes and CT volume using fixed SUV thresholds, isocontour thresholding relative to SUV_max_ (SUV%), thresholds relative to bone marrow (SUV_BM_), thresholds relative to parotid gland (SUV_parotis_) and thresholds relative to intrasellar pituitary gland (SUV_sella_).

**Table 1:** Correlation and Bland Altman bias and agreement with fixed SUV thresholds

Parameter	*r*-value	*r*^2^-value	Level of significance		Correlation Method	Bias ± SD	95% Limits of Agreement from - to
SUV 2.5	0.753	0.567	< 0.001		Spearman	-0.96 ± 1.4	-3.79–1.86
SUV 3	0.746	0.556	< 0.001		Spearman	-0.35 ± 1.3	-2.85–2.15
SUV 3.5	0.751	0.565	< 0.001		Spearman	0.10 ± 1.2	-2.23–2.43
SUV 3.75	0.749	0.560	< 0.001		Spearman	-0.29 ± 1.1	2.48–1.90
SUV 4	0.783	0.613	< 0.001		Spearman	0.50 ± 1.0	-1.55–2.53
SUV 4.5	0.705	0.496	< 0.001		Spearman	-0.76 ± 1.0	-2.65–1.14
SUV 5	0.695	0.483	0.001		Spearman	-0.95 ± 0.9	-2.71–0.82
SUV 10	0.416	0.173	0.077		Spearman	-1.93 ± 0.9	-3.77 - -0.09
SUV 15	0.329	0.108	0.169		Spearman	-2.21 ± 1.3	-4.70–0.28

**Table 2:** Correlation and Bland Altman bias and agreement with isocontour volumetric correlation (percentage of SUV_max_)

Parameter	*r*-value	*r*^2^-value	Level of significance	Correlation Method	Bias ± SD	95% Limits of Agreement from - to
Iso 10	-0.085	0.007	0.730	Spearman	12.23 ± 10.7	-8.73–33.20
Iso 15	-0.047	0.002	0.847	Spearman	8.34 ± 8.6	-8.56–25.25
Iso 20	0.012	0.000	0.960	Spearman	5.25 ± 8.9	-6.23–16.72
Iso 25	0.016	0.000	0.950	Pearson	3.14 ± 3.9	-4.48–10.76
Iso 30	0.151	0.023	0.538	Pearson	1.66 ± 2.5	-3.20–6.51
Iso 35	0.352	0.124	0.139	Pearson	0.75 ± 1.7	-2.58–4.08
Iso 40	0.391	0.153	0.098	Pearson	0.07 ± 1.5	-2.81–2.96
Iso 45	0.496	0.246	0.031	Pearson	0.36 ± 1.2	-2.00–2.73
Iso 50	0.304	0.092	0.206	Spearman	-0.73 ± 1.1	-2.82–1.37

**Table 3:** Background based volumetric correlations and Bland Altman bias and agreement with SUV_BM_

Parameter	*r*-value	*r*^2^-value	Level of significance	Correlation Method	Bias ± SD	95% Limits of Agreement from - to
400% SUV_BM_	0.674	0.454	0.002	Spearman	-0.66 ± 1.3	-3.15–1.83
375% SUV_BM_	0.667	0.444	0.002	Spearman	-0.53 ± 1.4	-3.18–2.12
350% SUV_BM_	0.682	0.465	0.001	Spearman	-0.38 ± 1.5	-3.27–2.50
200% SUV_BM_	0.747	0.558	< 0.001	Spearman	1.18 ± 2.4	-3.49–5.85
150% SUV_BM_	0.859	0.738	< 0.001	Pearson	-2.1 ± 2.2	-6.35–2.08
60% SUV_BM_	0.429	0.184	0.067	Pearson	22.3 ± 13.2	-3.53–48.07
50% SUV_BM_	0.439	0.193	0.060	Pearson	18.65 ± 11.6	-4.09–41.39
40% SUV_BM_	0.539	0.290	0.017	Spearman	15.32 ± 10.5	-5.18–35.81
30% SUV_BM_	0.670	0.448	0.002	Spearman	11.00 ± 7.4	-3.49–25.49
10% SUV_BM_	0.518	0.269	0.023	Spearman	8.06 ± 7.05	-5.75–21.87

#### Fixed SUV thresholds

The highest correlation between CT-derived volume and a fixed SUV threshold was achieved using a SUV of 4.0 (*r* = 0.783. r^2^ = 0.613. *p* < 0.001). The other tested, fixed SUV thresholds resulted in an over- or underestimation of the CT-derived volume (Table 1).

#### Isocontour relative to SUV_max_ (SUV%)

Results demonstrated the highest correlation using a PET volume isocontour of 45% SUV_max_ (*r* = 0.496. r^2^ = 0.246. *p* < 0.031). The other tested isocontours comprised lower correlation to the CT-derived volume (Table 2).

#### Thresholds relative to reference tissue (SUV_BM_, SUV_parotis_, SUV_sella_)

The highest association between CT-derived volume and a threshold relative to SUV_BM_ was achieved with a threshold of 150% SUV_BM_ (*r* = 0.859. r^2^ = 0.738. *p* < 0.001). Other tested thresholds revealed a lower association (Table 3).

The highest association between CT-derived volume and a threshold relative to SUV_parotis_ was found with a threshold of 250% SUV_parotis_ (*r* = 0.460. r^2^ = 0.212. *p* < 0.047). Other tested thresholds revealed lower association (Table 4).

**Table 4:** Background based volumetric correlations and Bland Altman bias and agreement with SUV_parotis_

Parameter	*r*-value	*r*^2^-value	Level of significance	Correlation Method	Bias ± SD	95% Limits of Agreement from - to
400% SUV_parotis_	0.432	0.186	0.065	Spearman	-0.50 ± 1.6	-3.71–2.71
375% SUV_parotis_	0.455	0.207	0.050	Spearman	-0.33 ± 1.7	-3.72–3.06
350% SUV_parotis_	0.436	0.190	0.062	Spearman	-0.14 ± 1.8	-3.76–3.47
300% SUV_parotis_	0.452	0.204	0.052	Spearman	0–28 ± 2.1	-3.91–4.48
250% SUV_parotis_	0.460	0.212	0.047	Spearman	-0.87 ± 2.5	-5.81–4.06
200% SUV_parotis_	0.302	0.091	0.208	Spearman	1.63 ± 2.9	-4.02–7.27
150% SUV_parotis_	0.281	0.079	0.247	Spearman	3.22 ± 4.1	-4.80–11.24
80% SUV_parotis_	0.046	0.002	0.850	Pearson	33.88 ± 22.0	-9.29–77.05
70% SUV_parotis_	0.450	0.202	0.053	Spearman	25.67 ± 15.6	-4.95–56.30
60% SUV_parotis_	0.418	0.175	0.075	Spearman	21.93 ± 13.4	-4.30–48.16
40% SUV_parotis_	0.326	0.106	0.173	Spearman	15.28 ± 10.4	-5.18–35.73

Thresholds relative to SUV_sella_: The highest association between CT-derived volume and a threshold relative to SUV_sella_ showed a threshold of 70% SUV_sella_ (*r* = 0.819. r^2^ = 0.670. *p* < 0.001). Other tested thresholds revealed lower association (Table 5).

**Table 5:** Background based volumetric correlations and Bland Altman bias and agreement with SUV_sella_

Parameter	*r*-value	*r*^2^-value	Level of significance	Correlation Method	Bias ± SD	95% Limits of Agreement from - to
125% SUV_sella_	0.564	0.318	0.012	Spearman	-1.87 ± 0.9	-3.69 - -0.06
80% SUV_sella_	0.593	0.352	0.007	Spearman	2.95 ± 3.4	-3.71–9.61
70% SUV_sella_	0.819	0.670	< 0.001	Spearman	-0.83 ± 1.9	-4.58–2.92
60% SUV_sella_	0.815	0.664	< 0.001	Spearman	-0.04 ± 1.6	-3.21–3.13
50% SUV_sella_	0.706	0.498	0.001	Spearman	-0.58 ± 1.4	-3.38–2.21
40% SUV_sella_	0.597	0.357	0.007	Spearman	-0.96 ± 1.3	-3.46–1.53
35% SUV_sella_	0.579	0.335	0.009	Spearman	-1.10 ± 1.1	-3.43–1.23
30% SUV_sella_	0.622	0.387	0.005	Spearman	-1.22 ± 1.1	-3.43–0.10

**Fig. 1:**
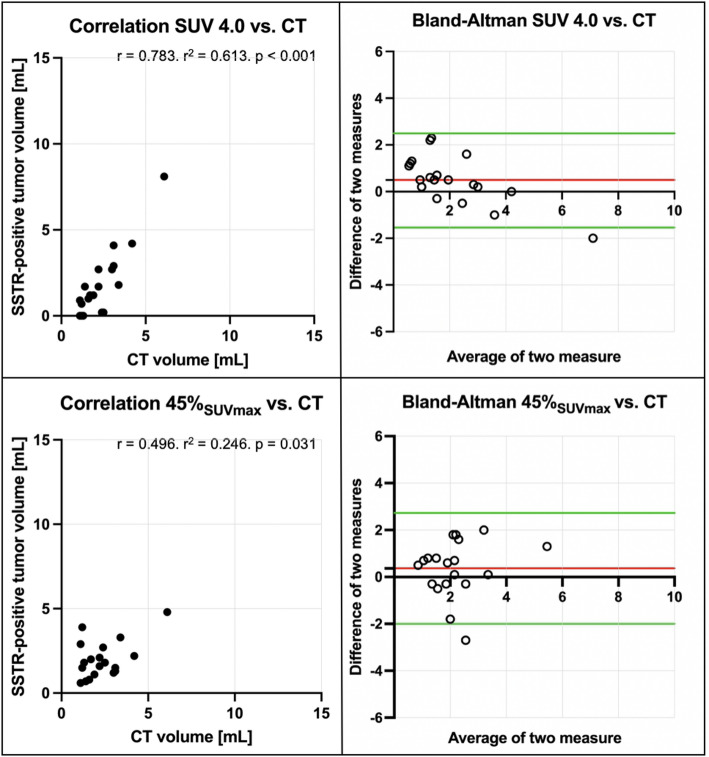
Correlation of PET volumes and CT-based reference standard. Upper row: PET volume SUV 4.0 (*r* = 0.783. r^2^ = 0.613. *p* < 0.001). Lower row: PET volume isocontour of 45% SUV_max_ (*r* = 0.496. r^2^ = 0.246. *p* = 0.031); each correlation plot is accompanied by the corresponding Bland-Altman plot (red line: mean difference of two measures; green lines: mean difference of two measures ± 1.96 * standard deviation of the mean difference

**Fig. 2:**
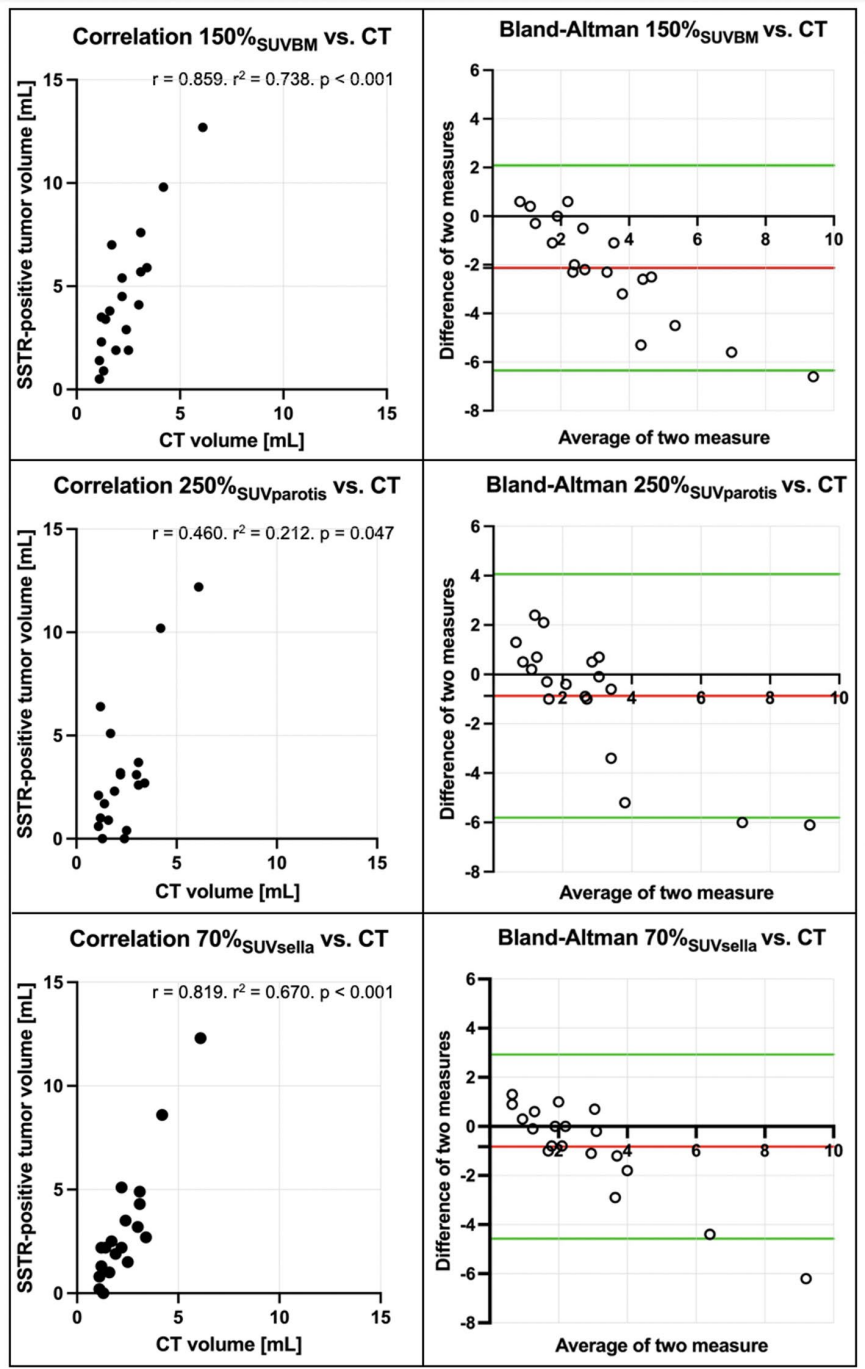
Correlation of PET volumes using background tissue and CT-based reference standard. Upper row: PET volume 150% SUV_BM_ (*r* = 0.859. r^2^ = 0.738. *p* < 0.001). Middle row: PET volume 250% SUV_parotis_ (*r* = 0.460. r^2^ = 0.212. *p* = 0.047). Lower row: PET volume 70% SUV_sella_ (*r* = 0.819. r^2^ = 0.670. *p* < 0.001); each correlation plot is accompanied by the corresponding Bland-Altman plot (red line: mean difference of two measures; green lines: mean difference of two measures ± 1.96 * standard deviation of the mean difference

As demonstrated in Fig. 1 and 2 correlation and Bland-Altman plots are employed to illustrate the optimal correlation between PET- and CT-derived volumes. An illustrative case is presented in Fig. 2.

**Fig. 3:**
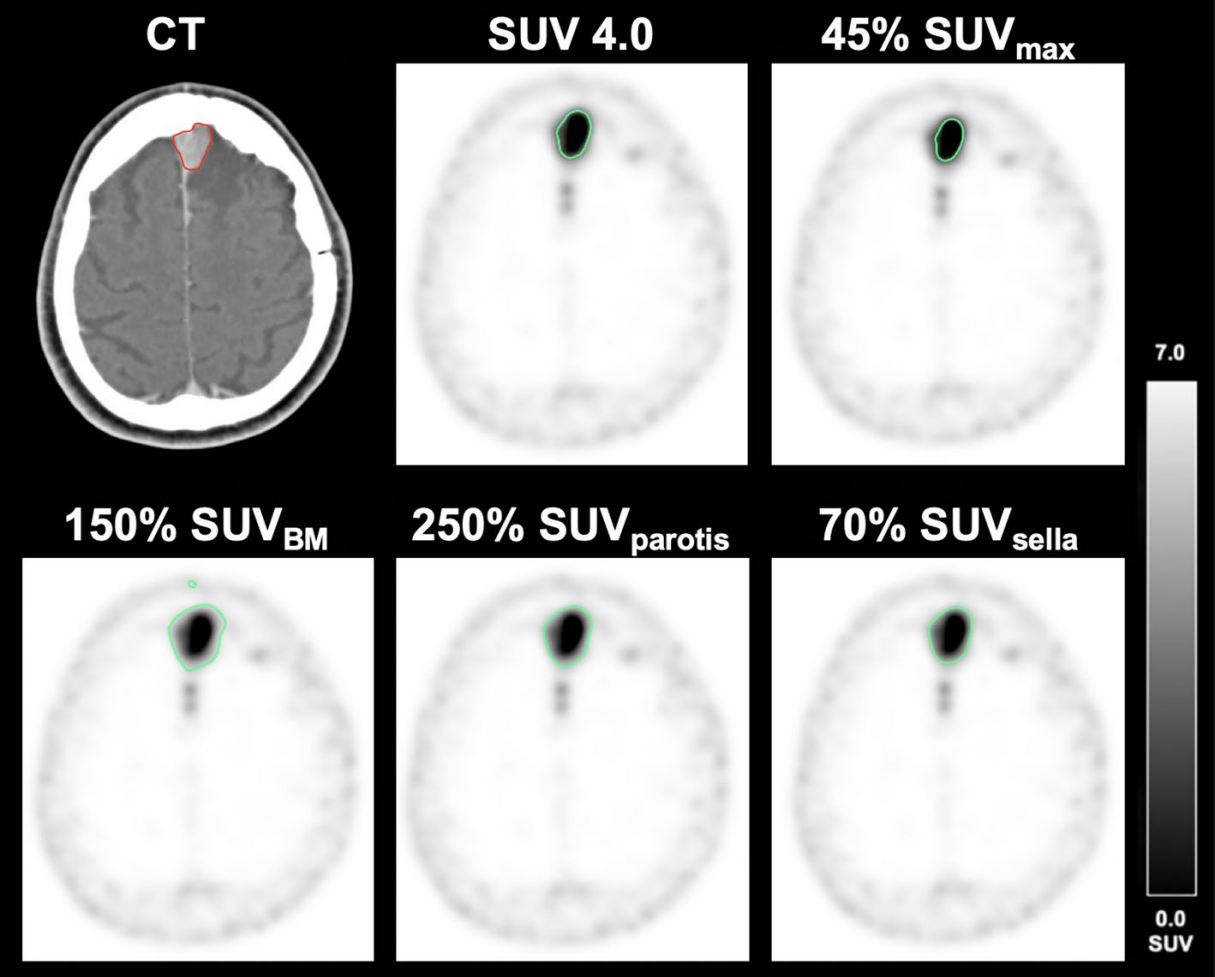
A 57-year-old male patient with meningothelial meningioma, CNS WHO grade 1 at the left falx. The meningioma was delineated applying the best threshold of the respective approach. Volumetric reference standard 4.2 mL; SUV 4.0: 4.2 mL; 45% SUV_max_: 2.2 mL; 150% SUV_BM_: 9.8 mL; 250% SUV_parotis_: 10.2 mL; 70% SUV_sella_: 8.6 mL

### [^18^F]SiTATE-avidity of background tissues

Highest SUV mean in reference tissues was found in the pituitary gland (mean 9.85 ± 2.8) followed by the bone marrow (mean 1.34 ± 0.3). The parotid gland showed lowest uptake (mean 1.33 ± 0.5). The resulting CoV was 24.4% using the bone marrow, 38.2% using the parotid gland and 28.0% using the pituitary gland. The corresponding [^18^F]SiTATE-avidity of the different background tissues with each other did not intercorrelate (Table 6).

**Table 6:** Correlation of background tissues SUV_BM_, SUV_parotis_ and SUV_sella_

Parameter	Bone marrow	Parotid gland	Intrasellar pituitary gland
SUVmean [median (range)]	1.26 (0.67–1.89)	1.42 (0.63–2.09)	9.64 (5.86–15.23)
Coefficient of variation (CoV)	24.4%	38.2%	28.0%
Correlation with bone marrow	-	*r* = 0.218 (*p* = 0.371)	*r* = 0.393 (*p* = 0.096)
Correlation with parotid gland	*r* = 0.218 (*p* = 0.371)	-	*r* = 0.270 (*p* = 0.263)
Correlation with intrasellar pituitary gland	*r* = 0.393 (*p* = 0.096)	*r* = 0.270 (*p* = 0.263)	-

### Individual backwards thresholding

The backwards thresholding revealed a corresponding fixed SUV threshold was 3.5 (mean; SD ± 1.39). The resulting CoV was 39.2%. However, results demonstrated a slightly lower correlation coefficient (*r* = 0.751, r^2^ = 0.565, *p* < 0.001, Table 7) compared to the analyses above after applying these resulting mean values of backwards thresholding to all 19 meningiomas and correlating these volumes to the CT-derived volumetric reference.

**Table 7:** Individual backwards thresholding

	SUV
Mean ± standard deviation	3.5 ± 1.39
Coefficient of variation (CoV)	39.2%
Correlation with CT reference (SUV 3.5)	*r* = 0.751
Coefficient of determination (SUV 3.5)	r^2^ = 0.565
Level of significance (SUV 3.5)	*p* < 0.001

## Discussion

Measuring the volumetric extent of meningiomas is of great interest, as it provides significant information for surgery or radiotherapy planning. Due to its many advantages over ^68^Ga-labeled SSTR-ligands, the ^18^F-labeled SSTR targeting peptide, [^18^F]SiTATE, might become increasingly important in meningioma PET imaging [1–7]. In direct comparison to ^68^Ga-labeled SSTR-ligands, Unterrainer et al. were able to show that the [^18^F]SiTATE determined tumor-to-background ratio is superior [5]. Furthermore, due to its ^18^F labeling, [^18^F]SiTATE offers improved spatial resolution, generally resulting in better intraosseous lesion detection [5]. Currently, the choice between these SSTR tracers primarily depends on their availability at the respective institution.

In this first analysis, we correlated meningioma volumes derived from different threshold-based approaches for PET-based delineation with the CT-derived volumetric reference using [^18^F]SiTATE. In this retrospective study, contrast-enhanced CT was used as the anatomical reference for volumetric correlation with PET due to its consistent availability across all included datasets. We acknowledge that, despite its routine integration into the SSTR PET/CT protocol, contrast-enhanced CT is inferior to MRI in terms of soft tissue contrast; however, recent data—particularly from the RANO/PET working group—have demonstrated the superiority of SSTR PET over MRI for accurate tumor delineation, especially in anatomically complex areas, such as periorbital area, or regions with low MRI contrast [13, 14]. With the intention of addressing the limitations in precisely assessing the full extent of meningiomas, we performed a volumetric determination of well-delineated lesions.

While it is not feasible to define a threshold that includes all meningioma cells due to the inherent physical limitations of PET imaging an optimal threshold can be established that approximates the true tumor extent as closely as possible [15, 16]: Partial volume effects can lead to underestimation of tracer uptake at the edges of the tumor, affecting threshold-based segmentation methods [15]. The uptake of SSTR-targeted tracers is dependent on receptor density (SSTRs) and tumor perfusion, as demonstrated for [^18^F]FDG, which can lead to variability in signal intensity [17, 18]. These factors must be considered when developing a robust segmentation approach. Our data implicate that a fixed SUV threshold of 4.0 is a practical and reliable option for tumor delineation (*r* = 0.783, r² = 0.613, *p* < 0.0001). While some individualized thresholds showed slightly higher correlation with the reference standard, such as 150% SUV_BM_ (*r* = 0.859, r² = 0.738, *p* < 0.0001), the use of a fixed threshold is simpler to implement in clinical practice and can be applied cross-institutional. In particular, a SUV is a commonly used and readily accessible metric in PET imaging, no specific software or algorithms are required to assess the extent of meningiomas on [^18^F]SiTATE PET/CT.

Even if the correlation of 150% SUV_BM_ and 70% SUV_sella_ with the CT-derived reference is higher than the correlation using a fixed SUV threshold of 4.0, the chance of bias increases, as e.g. the SiTATE-avidity differs within the bone marrow. Additionally, the reference tissues (i.e. bone marrow, parotid gland, pituitary gland) showed a high inter-individual variability with CoV values up to 38.2% and did not correlate with one another on an intra-individual level. Also, the avidity for SSTR tracers in the pituitary gland is inhomogeneous between different patients, as demonstrated for [^68^Ga]Ga-DOTA-TOC [20] or can be substantially underrated, e.g. in case of empty sella phenomenon or previous surgery. Additional analysis of the data visualized in Bland-Altman plots (see Figs. 1 and 2) showed best performance using a fixed SUV threshold of 4.0 for delineation. The 150% SUV_BM_ and 70% SUV_sella_ overestimated the PET-based volume in larger lesions (Fig. 3).

We also tried to derive an optimal threshold on a backwards step approach. However, the reverse deduction of a PET-based threshold is limited by the obtained dispersion of threshold values (CoV of 39.2%), when adjusting the threshold value to achieve the identical volume on PET. When applying the resulting mean SUV value to all meningiomas, correlation analysis with the CT-based reference resulted in an inferior correlation compared to the application of a fixed SUV threshold of 4.0. However, it must be noted that the number of meningiomas included was rather small. Thus, further studies are necessary to investigate, if the fixed SUV of 4.0 is superior compared to the backward-thresholding-derived value of 3.5. Nevertheless, it must be discussed that this approach cannot be applied to meningiomas without significant SiTATE-avidity.

Moreover, additional adjustment is required in case of close vicinity to areas of physiologically high SiTATE-avidity, e.g. the pituitary gland. Using a fixed SUV threshold would cause an unintentionally inclusion of these areas. Furthermore, this delineation approach is independent of the SUV_max_ value within the meningioma affecting an isocontour based approach. As also shown for other ligands [21], diverging PET-scanners and reconstruction algorithms do rather affect the reproducibility of SUV_max_ values than the mere SUV value, which is promising for an approach with high validity and reliability. However, further studies are needed to investigate the reproducibility of PET parameters on [^18^F]SiTATE-PET in patients with meningioma with emphasis on the influence of vendors and reconstruction algorithms.

Our analysis has several limitations that need to be addressed: especially the low number of patients must be mentioned, which limits the validity and statistical power of our analysis. While we applied Pearson or Spearman correlation coefficients based on the distribution of each variable as determined by the Shapiro–Wilk test, we acknowledge that the limited number of observations affects the robustness and comparability of correlation measures. In this context, non-parametric methods such as Spearman correlation were preferred whenever normality could not be reliably assumed. The results should therefore be interpreted with caution and considered exploratory in nature. Even though we only included meningiomas with a volume of at least 1.0 mL, there is the possibility that some of them might be susceptible to partial volume and spillover effects [15]. The variability in tracer uptake times represents a potential limitation, as it may have influenced SUV measurements. However, all scans were acquired under routine clinical conditions. Beyer et al. suggested an optimal scan time point at 120 min p.i., followed by 60 min p.i., based on a study with acquisition times ranging from 10 to 180 min over 15–20-minute intervals [22]. They reported that differences in uptake time primarily affect the tumor-to-background ratio—which was visually sufficient in all our cases—supporting the validity of our imaging window [22].

Our results need further verification for meningiomas with intraosseous extension, as they were excluded from this study. These results are based on preliminary data and require further validation. Ongoing studies aim to evaluate the applicability of this approach to intraosseous and mixed meningiomas. Most importantly, this study introduces a methodology for meningioma delineation that provides a foundation for future research and requires further validation. Additional studies are needed to compare the performance of the proposed delineation approach with established delineation approaches in ^68^Ga tracer imaging. Furthermore, larger cohorts and studies including correlation between histopathology and tracer uptake are planned to confirm and refined the proposed threshold.

## Conclusion

A fixed SUV threshold of 4.0 for delineation of meningioma showed strong association with the CT-derived volumetric reference in this preliminary analysis. By using this approach results are both less prone to error in case of changes of [^18^F]SiTATE-avidity in background tissues (e.g. bone marrow) and easily applicable in clinical routine without specific technical or software requirements. Complementary studies applying this approach for volumetric delineation of meningiomas are intended.

## Supplementary Information

Below is the link to the electronic supplementary material.

Supplementary Material 1

## Data Availability

The datasets used and/or analyzed during the current study are available from the corresponding author on reasonable request.

## Abbreviations

CoV: Coefficient of variation
CT: Computed tomography
NET: Neuroendocrine tumor
PET: Positron-emission tomography
ROI: Region of interest
SD: Standard deviation
SSTR: Somatostatin receptor
VOI: Volume of interest
