# Supplementary material for: Feasibility of different meningioma delineation approaches on [18F]SiTATE PET/CT imaging
Source: Discov Oncol. 2025 Nov 14;16:2189. doi: 10.1007/s12672-025-04079-6 (PMC12669456; doi:10.1007/s12672-025-04079-6)
Supplement: Supplementary file 1 — Supplementary Material 1 [file 12672_2025_4079_MOESM1_ESM.docx]

**Supplementary Material:** Extended patients’ characteristics

|  | | **Patient characteristics** | | | | | **Technical information** | | **Volumetric information** | | | | | | |
| --- | --- | --- | --- | --- | --- | --- | --- | --- | --- | --- | --- | --- | --- | --- | --- |
| **No.** | **Sex** | **Tumor localization** | **WHO grade** | **Histologic subtype** | **Local pretreatment** | **Age** | **Activity [MBq]** | **Acquisition time [min]** | **Volume meningeoma** | **SUV_max_ meningeoma** | **Volume SUV 4.0** | **45% SUV_max_** | **150% SUV_BM_** | **250% SUV_parotis_** | **70% SUV_sella_** |
| 1 | F | posterior sinus sagittalis | 2 | n.a. | None | 69 | 170 | 79 | 2.2 | 4.6 | 2.7 | 2.1 | 5.4 | 320 | 5.10 |
| 2 | M | falcine | 1 | meningothelial | None | 57 | 139 | 54 | 4.2 | 4.05 | 4.2 | 2.2 | 9.8 | 10.2 | 8.60 |
| 3 | M | frontal | n.a. | n.a. | None | 57 | 228 | 55 | 6.1 | 6 | 8.1 | 4.8 | 12.7 | 12.2 | 12.30 |
| 4 | M | frontal | 1 | n.a. | None | 17 | 167 | 60 | 2.4 | 2.25 | 0.2 | 2.7 | 2.9 | 0 | 3.50 |
| 5 | F | cerebellar tentorium | 1 | microcystic | None | 52 | 196 | 64 | 2.2 | 3.1 | 1.7 | 1.6 | 4.5 | 3.1 | 2.20 |
| 5 | F | parafalcine | 1 | microcystic | None | 52 | 196 | 64 | 1.7 | 3.7 | 1.2 | 2 | 7 | 5.1 | 2.50 |
| 6 | F | olfactory nerve | n.a. | n.a. | None | 58 | 263 | 60 | 1.2 | 3.2 | 0.7 | 1.5 | 3.5 | 1 | 2.2 |
| 7 | F | falcine | 1 | meningothelial | None | 70 | 114 | 77 | 3.4 | 2.9 | 1.8 | 3.3 | 5.9 | 2.7 | 2.7 |
| 8* | F | cerebellar tentorium | 1 | microcystic | None | 52 | 141 | 85 | 3.0 | 3.6 | 2.7 | 1.2 | 4.1 | 3.1 | 3.2 |
| 9 | F | temporal | n.a. | n.a. | None | 76 | 170 | 95 | 1.3 | 1.95 | 0 | 1.8 | 0.9 | 0 | 0 |
| 10 | F | frontobasal | n.a. | n.a. | None | 58 | 156 | 63 | 1.6 | 2.9 | 1 | 0.8 | 3.8 | 0.9 | 1 |
| 11 | F | parietal | 2 | atypic | None | 50 | 144 | 92 | 2.5 | 1.8 | 0.2 | 1.8 | 1.9 | 0.4 | 1.5 |
| 12 | F | parafalcine | 1 | transitional | None | 46 | 149 | 93 | 1.2 | 3.25 | 0 | 3.9 | 2.3 | 6.4 | 1.3 |
| 12 | F | parafalcine | 1 | transitional | None | 46 | 149 | 93 | 1.1 | 2 | 0 | 2.9 | 0.5 | 2.1 | 0.2 |
| 13 | M | frontal | 2 | atypic | None | 63 | 130 | 102 | 3.1 | 3.7 | 2.9 | 1.5 | 5.7 | 2.6 | 4.3 |
| 14 | M | cerebellar tentorium | 1 | transitional | None | 65 | 129 | 92 | 1.4 | 5 | 1.7 | 0.7 | 3.4 | 1.7 | 2.2 |
| 15 | F | temporoparietal | 2 | atypic | None | 42 | 136 | 96 | 3.1 | 7.2 | 4.1 | 1.3 | 7.6 | 3.7 | 4.9 |
| 16 | F | frontal | n.a. | n.a. | None | 43 | 134 | 104 | 1.9 | 2.7 | 1.2 | 1.1 | 1.9 | 2.3 | 1.9 |
| 17 | F | frontoparietal | n.a. | n.a. | None | 80 | 211 | 157 | 1.1 | 3.24 | 0.9 | 0.6 | 1.4 | 0.6 | 0.8 |

*Patient No. 5 received a follow up PET/CT scan; n.a. not applicable (no histology or report available); F female; M male, n.a. not applicable
